# The Relevance of a Physiological-Stage Approach Study of the Molecular and Environmental Factors Regulating Seed Germination in Wild Plants

**DOI:** 10.3390/plants10061084

**Published:** 2021-05-28

**Authors:** Ximena Gómez-Maqueo, Laura Figueroa-Corona, Jorge Arturo Martínez-Villegas, Diana Soriano, Alicia Gamboa-deBuen

**Affiliations:** 1 Instituto de Ecología, Universidad Nacional Autónoma de México, Mexico City 04510, Mexico; ximenagmb@ciencias.unam.mx (X.G.-M.); laurafc25@gmail.com (L.F.-C.); jorge.mtzvillegas@ciencias.unam.mx (J.A.M.-V.); 2 Facultad de Ciencias, Universidad Nacional Autónoma de México, Mexico City 04510, Mexico; dsoriano@ciencias.unam.mx

**Keywords:** germination, dormancy, non-model species, wild species, plant domestication, seed physiology, seed maturation

## Abstract

Germination represents the culmination of the seed developmental program and is affected by the conditions prevailing during seed maturation in the mother plant. During maturation, the dormancy condition and tolerance to dehydration are established. These characteristics are modulated by the environment to which they are subjected, having an important impact on wild species. In this work, a review was made of the molecular bases of the maturation, the processes of dormancy imposition and loss, as well as the germination process in different wild species with different life histories, and from diverse habitats. It is also specified which of these species present a certain type of management. The impact that the domestication process has had on certain characteristics of the seed is discussed, as well as the importance of determining physiological stages based on morphological characteristics, to face the complexities of the study of these species and preserve their genetic diversity and physiological responses.

## 1. Introduction

The seed plays a fundamental role in the plant life cycle as a “pause” stage between successive plant generations [1]. It also functions as a resistance structure to adverse environmental conditions and as a unit for dispersal of individuals in both time and space [1]. Thus, plant populations depend on the success of a series of biological processes that are regulated by environmental conditions and that will impact on the germination process and the seed-seedling transition [2,3]. These biological processes modulated by the environment include genetic and developmental programs such as seed maturation, dormancy, longevity, timing of seed release, and germination, but also include other factors such as dispersal strategies and the effect of biotic and abiotic interactions on germination and during the seed-seedling transition [3,4,5].

In angiosperms, the seeds’ constituents include an outer cover or testa, the endosperm and the cotyledon or cotyledons, and the embryonic axis [1]. The characteristics of each component and the relationship between each component are highly variable between the seeds of different species [1]. In dicots, the endosperm may be present or absent in the dry, mature seeds. Endospermic seeds can have one or more cell layers of this tissue surrounding the embryo, as in the case of *Arabidopsis thaliana* (L.) Heynh (Arabidopsis), and, in the case of species with folded cotyledons, the endosperm may be located in-between the folds. In general, the endosperm is abundant in monocots [6]. In dicots, there are about 10 different types of embryo morphologies, five of which does not occur in monocots [7]. In general, the endosperm is very abundant in monocots due to a smaller embryo size with respect to total seed size [6,7]. Additionally, there are six types of embryos present in monocots, two of which are exclusive to the monocot clade. One of the most known embryo types is the lateral embryo type, which occurs in the Poaceae family. In gymnosperms, the embryo can be linear and small in proportion to the total seed size, or spatulate and proportionally large in comparison with total seed size [7].

Embryogenesis is characterized by cell division and organ formation. The time required for the development of a seed is regulated by multiple environmental factors. Therefore, chronological age is a less adequate approximation than physiological age. Physiological age has been determined from easily quantifiable characteristics such as weight, size and color, in addition to a series of species-specific morphological and anatomical characteristics [8]. 

In general, the embryogenesis concludes and embryo growth ceases during seed maturation before seed shattering. In some plant species, the embryogenesis and maturation finish after seed dispersion. At the maturation stage, in which major reserve substances accumulate, the desiccation tolerance and dormancy status are acquired. These processes have been extensively reported to occur in the so-called orthodox seeds, which enable them to persist for long periods in naturally occurring soil seed banks, as well as in ex situ seed banks. Orthodox seeds can be quiescent or dormant. Quiescent seeds have very little metabolic activity that is activated to germinate in the appropriate environmental conditions, mainly determined by the availability of water to hydrate the seed and by temperature [1]. Dormant seeds, where the depth of dormancy is determined by environmental conditions during maturation, present special requirements for germination [9]. Recalcitrant seeds occur among different taxa and can experience a slight dehydration process during seed maturation, but are sensitive to further desiccation below a species-specific threshold and cannot be stored for prolonged periods. In natural populations, these seeds may germinate within the fruit or soon after dehiscence, and they do not persist in the soil seed bank. These types of embryos rapidly lose viability if they are dried or chilled. The term recalcitrant is generally applied to seeds that have been systematically tested to determine their ability to tolerate desiccation [10]. Germination *sensu stricto* refers to the events that begin with the hydration of the mature seed and the elongation of the radicle until its protrusion takes place [1].

This article will review the molecular bases of different processes involved in the recruitment of new individuals from a community, including the effect of environmental conditions, with special emphasis on non-model plants. It is important to point out that the use of physiological stages rather than chronological age would benefit the elucidation of the molecular and physiological mechanisms involved in seed germination performance and seedling establishment. This approach also aims to achieve a proper contrast between plant populations across genotypes, cohorts, localities and experimental treatment.

## 2. Molecular Bases of Seed Maturation

The different processes that occur during seed maturation are related to storage products accumulation, desiccation tolerance and seed dormancy. Seed maturation is highly regulated by the parental environmental cues such as temperature, light, nitrate and water [9].

Stressful environmental conditions during seed development also affect seed germination. *Glycine max* (L.) Merr. (soybean) seeds that were developed in plants subjected to heat stress showed a decrease in the speed of germination and, in the three determined stages (dry) seed, 6 h of imbibition and germinated, an increase in the detection and expression of genes related to abiotic stress was also detected [11].

### 2.1. Accumulation of Reserves

The molecular and physiological mechanisms of reserve substance storage in the seed have been extensively studied in Arabidopsis and in dicots such as legumes, and in monocots. The transcription factors FUSCA, ABI3 LEC1 and LEC2 (“LAFL” network) participate in the acquisition of desiccation tolerance, and reserve accumulation has been established by means of transcriptomic studies and the use of mutants [12]. Recently, it was described that the ABI3 homologous-gene in *Arachis hypogaea* L. (peanut) is also involved during seed filling under dark conditions [13]. This network was also described in gymnosperms [14]. The synthesis and accumulation of reserve proteins is similar in different plant species regardless of the botanical group (dicotyledonous or monocotyledonous, in angiosperms or gymnosperms), the storage behavior (orthodox or recalcitrant) [1] or the life history (perennials or annuals/biennials).

The accumulation of starch in the endosperm of the Poaceae family has been extensively studied in maize mutants, and important differences have been found with that of Arabidopsis. Loss of function of the GW2 and qSW5 genes (encoding a RING-type E3 ubiquitin ligase of unknown function, and a nuclear protein of unknown function (respectively) are involved in seed size increase in *Oryza sativa* L. (rice) [15,16]. These two proteins are involved in the positive regulation of GS3, a transmembrane protein with a cysteine-rich domain that participates in the negative regulation of seed size [17].

### 2.2. Tolerance to Desiccation

High tolerance to desiccation, associated to an important decrease on water content, was described in seeds for a wide variety of plant species [18]. Orthodox seeds can withstand a water content between 5% and 15% of total seed weight. Recalcitrant seeds usually present a high-water content at seed maturity, between 35% and 60% of total seed weight, indicating that this type of seed is also subject to stress during seed maturation [18]. Between 7.5% and 19.6% of the world’s plant species present seeds that are sensitive to desiccation [19]. These plants are present in tropical environments, temperate regions and semi-arid ecosystems [20]. Tolerance to desiccation is a complex trait that is under the control of different genes, but potentially small changes would be needed to eliminate this trait and make the seeds of a species sensitive to desiccation. This characteristic may have appeared, independently, several times in plant evolution [21].

Different processes or mechanisms were described as important factors in tolerance to dehydration [20]. The factors identified include the accumulation of protective molecules, such as LEAs, sucrose and oligopolysaccharides, such as raffinose and the “switching off” of metabolism including cell cycle activity [20].

The LEA proteins are present in both orthodox and recalcitrant seeds, as well as in angiosperms and gymnosperms [22,23]. A comparative study of LEA proteins involved in seed function between recalcitrant seeds of *Castanospermum australe* A.Cunn. and C. Fraser (Moreton Bay chestnut or blackbean) and orthodox seeds of *Medicago truncatula* Gaertn. (barrelclover) indicated that both types of seeds present four common LEA proteins with similar levels, but 12 were less abundant in blackbean seeds. These results suggest that the developmental program involved in desiccation tolerance is present in both types of seeds [22]. The accumulation of sucrose and raffinose is also involved in desiccation tolerance. The presence of transcripts involved in the synthesis of these saccharides was described in mature seeds with either orthodox or recalcitrant behavior. The reduction of metabolic activity is also essential for seed tolerance to desiccation. In orthodox seeds, there is an important decrease in the expression of transcripts involved in energy production and respiration [24]. In orthodox seeds, most cells are arrested in the G2 phase and DNA replication, one of the first events to arrest during dehydration. Recalcitrant seeds have an active metabolism when they are dispersed. Consequently, germination can potentially start, and end, without external water. In recalcitrant seeds of *Avicennia marina* (Forssk.) Vierh. (gray mangrove), a transient arrest of replication was detected no more than 24 h prior to release from the mother plant and germination [20].

The evergreen oak *(Quercus ilex* L.) is one of the most representative trees in Mediterranean forests and presents recalcitrant seeds. Dehydrin accumulation was reported in mature seeds, which are desiccated up to 38%. Additionally, there is an important accumulation of transcripts related to raffinose synthesis in both evergreen and northern red oak (*Q. rubra* L.) [25,26]. In order to study the maturation and germination processes as a continuum, a proteomic study was carried out in acorns at different stages of development (early development M4–M6 and late development M8–M9, where M9 corresponds to the mature acorn) and during germination (early G1–G3 and late G4–G5). These stages were determined based on morphology, weight and water content (the lowest water content was 60% and 35% in the embryo and cotyledons, respectively, in the M9 stage). Proteomic analysis of the different stages corresponding to maturation and germination showed that in these seeds there was a metabolic continuum in the embryo between the late stages of maturation and early germination. These two stages share a highly similar proteomic profile suggesting that there is no metabolic switch between them. As it was described, the recalcitrant seeds are released with an active metabolism, possibly associated with their sensitivity to dehydration [27].

## 3. Molecular Bases of Dormancy

Dormancy refers to a state that is characterized by a temporary arrest of the growth and development of a plant or some of its organs. In seeds, the definition implies the absence of germination in a viable, hydrated, seed that is in favorable conditions for germination [1,28]. Dormancy is a trait that arose in many species belonging to different taxa through their evolutionary history by natural selection in environments that present unfavorable conditions such as heat, cold or drought [1]. The number of species that present dormant seeds tends to increase with geographical distance from the equator, as seasonal variation in the pattern of precipitation and temperatures increases [7]. The biological function of dormancy is mainly to disperse germination in time, which can be achieved in two ways. The first one deals with the dependence of dormancy breakage on environmental factors that, in turn, are distributed differently through the time of year. This is one of the most known mechanisms of dormancy, as it implies the requirement of an environmental factor such as a chilling period during winter for summer annuals to germinate and develop during the most favorable season [1,29]. Within a seed population, different depths of dormancy can be detected across individual seeds, but in some species these differences are more intense, leading to the second type of temporal dispersal, through the expression of polymorphisms, heteromorphy or heteroblasty. In this type of dormancy, the differences in morphology and degree of dormancy are distinguishable between seeds of the same plant or among plants of the same species. Examples of this phenomenon are mainly registered in the Asteraceae and Chenopodiaceae families and allow for different portions of the population to germinate at different times [30]. Recent studies, using two different germplasms of *Leymus chinensis* (Trin.) Tzvelev (sheepgrass) with high and low germination performance, suggest that the level of ABA at the middle and late maturation stages is involved in the germination performance [31].

Finally, a non-dormant seed is one which can germinate under the widest range of environmental conditions that are compatible with the germination requirements for a particular species [4,32].

There are several classifications and sub-classifications of dormancy based on the various mechanisms by which the block of germination can occur. The most common types of dormancy reported in the literature are physiological dormancy, morphological dormancy, morphophysiological dormancy and physical dormancy. If based on the time at which dormancy is imposed, there are two main types of dormancy: primary dormancy, which occurs as part of seed development to prevent germination in the mother plant and to favor dispersal over a longer period after its detachment from the mother plant [32,33]; and secondary dormancy, which is a state induced in a mature seed during imbibition, as a consequence of adverse environmental conditions and that can usually be expressed in seeds with non-deep physiological dormancy [1,32]. Here, we present a short description of the main types of dormancy reported in the literature. However, these types have been known to occur in different combinations in some species.

Physical dormancy. For some species, the seed coat is impermeable to water and/or gas exchange, thus the germination process is blocked. The seed coat impermeability is usually associated with the presence of one or more layers of impermeable palisade cells, and the natural openings of a seed, such as the micropyle, hilum and chalazal area, also become impermeable to water. Specialized openings, valves or plugs have evolved, and it is not until these plugs are dislodged, usually by fluctuations in soil temperature, that water can enter the seed. In some cases, ingestion by animals can also promote germination of this type of seeds [7,34]. Physical dormancy has not been described for gymnosperms but was reported in at least 18 families of angiosperms, but not all members of a family present this type of dormancy [7,35]. These families include members of the Cucurbitaceae, Malvaceae, Fabaceae, Nelumbonaceae, Rhamnaceae and Sapindaceae, among others [35].

Physiological dormancy. This is one of the most widespread and abundant type of dormancy, it deals with a hormonal regulation that prevents germination, and it is present in gymnosperms and major angiosperm clades. It is most common in species of temperate climates and in the model species in which dormancy and germination are usually studied, which include Arabidopsis, *Solanum lycopersicum* Lam. (tomato), *Nicotiana tabacum* L. (tobacco) and several cereals [4]. In species such as Arabidopsis, the depth of dormancy has been demonstrated to be affected by temperature, by nitrate deficiency, as well as by light intensity and photoperiod [9,36]. Additionally, the effect of the prevalent maturation environment was higher than the effect of the genotype [9].

Morphological dormancy. In some species, mature seeds contain a fully differentiated, but underdeveloped, embryo. In others, the embryo did not complete the process of histodifferentiation and, thus, needs to develop after its detachment from the mother plant. In either situation, the embryo needs to grow in order to protrude from the enclosing layers, and germination is delayed by this process. This type of dormancy occurs in many plant families from both temperate and tropical regions. Some representative families include Amborellaceae, Cycadaceae, Ginkgoaceae, Hydrophyllaceae, Liliaceae, Loranthaceae, Magnoliaceae, Orchidaceae, Orobancheaceae, and Ranunculaceae, among others [7].

Morphophysiological dormancy. This type of dormancy usually occurs in species with rudimentary or linear embryos in combination with a physiological block to germination. Thus, two processes must take place during germination: (1) embryo differentiation and growth, and (2) dormancy alleviation. Baskin and Baskin [7] reported that the order in which both processes occur may be species-specific.

Hilhorst [28] discussed that dormancy is not an all-or-nothing property of the seed, but a relative phenomenon whose expression varies with the environment. He pointed out some considerations that should be taken in the implementation of the term dormancy. The term depends on the determination of the “absence of germination”, which is intrinsically associated with germination time: a seed can be classified as dormant or non-dormant depending on the (arbitrary) time of germination measurement. A second situation stems from the characterization of “the appropriate conditions for germination”. When dormancy is characterized in a particular species, erroneous conclusions can be drawn if the analysis is performed within or outside the optimal temperature range or other species-specific requirements. Finally, it is important to understand that the different sub-classifications of the two most common types of primary dormancy, physiological and morphophysiological, have their usefulness in the conceptualization of questions from a phylogenetic, biogeographic or evolutionary perspective. However, they are not suitable for physiological and molecular studies since they are mostly arbitrary, and do not distinguish the underlying mechanisms that drive the expression of the dormancy status. This is particularly relevant since the criteria for determining the level of dormancy are subjective.

### 3.1. Regulation of Dormancy: The Role of Hormones and Dormancy-Specific Regulators

Seed dormancy is an important adaptive trait in the early-life history of a particular species, and it is modulated by endogenous and environmental factors [32,37]. The role of model species such as Arabidopsis and other members of the Brassicaceae family has been fundamental for the understanding of the regulation mediated by the ABA/GA balance and antagonism, and its integration with other dormancy-specific regulators [38,39,40]. These specific regulators include the proteins Delay of Germination 1 (DOG1), and Reduced Dormancy 5, which are reviewed by Chahtane et al. [32], and Nee et al. [39]. The current model of the integration of the dormancy status requires both ABA and DOG1, the absence of either one leads to the loss of dormancy [37]. The ABA signaling and the DOG1 pathway converge in the inhibition of the activity of the clade A PP2C phosphatases, which regulate downstream responses that will determine whether a seed germinates or not [37]. The DOG1 gene has been studied in other species such as *Triticum aestivum* L. (wheat) [41], and *Lactuca sativa* L. (lettuce) [42], but a protein blast in the NCBI database indicates that the DOG1 and DOG-like genes have been identified in the assembled genomes available for a wider range of species, including members of the Cleomaceae, Euphorbiaceae, Malvaceae, and Rubiaceae families [43,44]. Further studies of the signaling pathway mediated by DOG1 are required in non-model species.

The participation of ABA in the induction and maintenance of dormancy has been extensively studied in different plant species [45,46]. ABA biosynthesis is enhanced in seeds with deeper dormancy phenotypes, as observed in Arabidopsis Cape Verde Island ecotype, where an important increase in genes involved in biosynthesis of ABA, such as NCED, was described during dormancy induction (reviewed in [4]). Additionally, dormancy imposition and maintenance has been observed to depend on an intrinsic balance between both the synthesis and catabolism of GA and ABA, which will determine the dominance of either hormone and its downstream signaling cascades [4,47]. The early ABA perception and signaling pathway requires the participation of three main components: the ABA receptors (PYR/PYL/RCAR); the SnRK2s protein kinases; and the PP2Cs protein phosphatases [40]. The receptors and the SnRK2.2, 2.3, and 2.6 are positive regulators of ABA signaling. The receptors bind and inhibit the activity of the PP2Cs (negative regulators of ABA signaling). This, in turn, allows for the activation of the SNRKs, which phosphorylate downstream regulators such as ABI5 and the AREB/ABFs transcription factors of the bZIP class [40]. ABI5 functions are the main inhibitor of germination [40,48]. The ABI3 transcription factor is known to physically interact with ABI5, and this interaction is important for the synergistic activation of gene expression [48]. Another positive regulator of ABI5 expression during germination is the ABI4 transcription factor, which, in turn, represses lipid breakdown in the embryo [40].

Loss of dormancy is a typical domestication syndrome that allows for the majority of the seeds in a batch to germinate upon imbibition and with little input from other stimuli. The specific genes related to loss of dormancy have been described in several models including rice, soybean, and barley. The selection of a dormancy gene during the domestication process in the Poaceae family was also described. The loss of function of the G gene, which encodes for a CAAX-amino-terminal protease involved in the regulation of ABA synthesis, determines the loss of dormancy in Poaceae, Solanaceae and legume crops [49]. Dormancy loss during domestication and cultivar management could result in a viviparous phenotype commonly known as preharvest sprouting (PHS, [50]). The control of PHS is a result of a complex signaling network that includes internal and external cues. In cereals, ABA metabolism and signaling play an important role in PHS regulation. The involvement of GA and other phytohormones, carbohydrate metabolism, reactive oxygen species (ROS), nitric oxide (NO) and microRNAs were also demonstrated [51]. Many cultivars of *Chenopodium quinoa* C.L. Willdenow (Willd.) (quinoa) are susceptible to PHS, so the effect of an exogenous ABA treatment on germination and gene expression was studied. The expression of the transcription factors from B3, bZIP, GATA and LBMD families, involved in ABA mediated transcriptional regulation during seed dormancy and germination, was increased [52].

The dormancy release process depends on seed structure or composition and environment conditions. For example, it was described that ethylene is involved in dormancy release by overcoming the inhibitory release of ABA in different plant species [53]. Alternating temperatures are also relevant for dormancy release in some plant species. This procedure inhibits ABA synthesis and decreases ABA sensitivity. In *Cynara cardunculus* L. *var. sylvestris* Lam. (wild cardoon), alternating temperatures during imbibition increase germination and there is a stimulation of the expression patterns related with ethylene and ROS signaling. ABA catabolism and cell wall loosening was also increased by alternating temperatures [54].

### 3.2. Dormancy Break by Different Treatments

For the study of germination processes in wild plant seeds, an important obstacle can be the depth and type of dormancy. The different treatments to break dormancy can include stratification, the use of hormones such as GA3, and the use of chemicals such as NO and ethanol. In recent years, the effect of these treatments at the transcriptome level has begun to be studied to understand the mechanisms behind dormancy breakage. The mature seeds of *Paris polyphylla* Sm. (love apple) present a globular-stage embryo with morphophysiological dormancy. No germination occurs up to 18 months, but dormancy can be broken by stratification treatment. The participation of ABA in dormancy was described and high levels of ABI5 were detected in dormant seeds [55].

A transcriptomic analysis to determine the mechanisms related to primary dormancy release by stratification was performed in *Cunninghamia lanceolata* (Lamb.) Hook (chinese fir) seeds. The induction of a secondary dormancy using a heat treatment was also studied. These two processes are highly regulated by ABA/GA balance in this species. For primary dormancy release, an important decrease in the expression of RGA, a gene related to negative regulation to GA sensitivity was detected. In contrast, an important increase in the expression of ABA1, a gene related to ABA synthesis, was observed during secondary dormancy induction [56].

The seeds of *Ginkgo biloba* L. (ginko) are recalcitrant and have morphophysiological dormancy. The freshly released seeds present an embryo at heart-stage and require a cold and wet stratification process to complete development, and for the seed to germinate. For the transcriptomic study, three stages of embryo development were selected: induction of dormancy, maintenance of dormancy and release of dormancy. The comparison between transcriptomes indicates that ABA and GA3 are highly involved in dormancy maintenance and release, respectively. The phytohormones auxin and brassinosteroids (BR), associated to morphological development of the embryo, are also involved [57].

The seeds of *Fraxinus hupehensis* S.Z.Qu, C.B.Shang and P.L.Su (ash tree) present dormancy, and no germination was observed during the first year. Differential expression analyses between germinated seeds treated with GA3 and without treatment suggest that GA3 increased the production of energy. Key genes involved in the glycolytic pathway are strongly expressed in the germinated seeds with ethanol treatment [58].

For *Aconitum heterophyllum* Wall. ex Royle (atish), that grows at altitudes up to 4000 m above sea level, the seeds present morphophysiological dormancy which can be broken with an ethanol treatment. The proteomic analyses indicated that ethanol induces the germination of these seeds by increasing the synthesis of GA via LYTB-like protein and a probable acetyl-CoA acetyl transferase 2 like protein, proteins detected only in the seeds treated with ethanol. The results suggest ethanol treatment enhanced the growth potential of embryonic axis through an induction of metabolism and cell wall remodeling [59].

## 4. Molecular Bases of Germination

Arabidopsis, tomato and cereal crops have been the most studied plants for the elucidation of the germination process at the molecular level. The state-of-the-art of the molecular bases of germination can be consulted in Carrera-Castaño et al. [40]. Therefore, the majority of the knowledge that we now have about germination comes from annual plants. In perennial plants, these types of studies were carried out in: the poplar tree (*Poplar simonii* Carrière × *Poplar nigra* L. [60]); in cultivated species with recalcitrant seeds, such as the evergreen oak [27]; and, more recently, in wild tree species, such as *Ceiba aesculifolia* (Kunth) Britten and Baker f. (pochote) [61], and wild orchids, such as *Calanthe tsoongiana* Tang and F.T. Wang [62].

A chronological approach has been extensively used to study the germination process at a molecular level in model plants. However, during Arabidopsis seed germination, it was demonstrated that there are two distinct morphological stages at 25 h of imbibition. At this time point, 50% of seeds presented a ruptured testa stage associated with a distinct transcriptomic profile in contrast to the other 50% of imbibed seeds, which did not have a ruptured testa yet [63]. This approach, based on morphology rather than chronological time, has been frequently applied to study germination in the seeds of several species of orchids, but also in trees such as poplar and pochote [60,61,64]. In the tropical tree pochote, a physiological stage approach instead of a chronological progression of germination was implemented by sampling seeds at specific relative water contents (rwc), and morphological cues such as testa rupture and radicle protrusion. This approach allowed the authors to describe and track the transcriptional changes that occur in seed batches collected in different years, and with distinct germination performance, thus detecting the main molecular processes associated to the germination performance observed across phenotypes, and in response to pre-germinative treatments such as priming [61].

The desiccation of the seed and the subsequent rehydration causes damage to the different macromolecules contained within. The imbibition of the seed promotes the reactivation of different DNA and protein repair mechanisms. Damage to DNA, mainly by strand breakage, affects the viability of the seeds. In the first transcriptomic phase described for Arabidopsis, there is an upregulation of the genes related to the DNA repair process, such as DNA ligases and DNA polymerases [5,63]. The seed repair mechanisms that allow proper genome maintenance was extensively reviewed by Waterworth et al. [65].

It has been determined that the mature seeds of different plant species present about 12,000 to 18,000 stored transcripts in several species including Arabidopsis, rice, and pochote [61,63,66], some of which can be subject to degradation during the early stages of imbibition. These transcripts usually correspond to genes expressed during the development and maturation of the seeds.

Likewise, during the start of water intake, the transcription of genes related to the activation of primary metabolism, including the metabolism of carbohydrates, amino acids and hormones, as well as the genes involved in the modifications of the cell wall, have been detected. In recent years, it was also described that the alternative editing of these transcripts plays a very important role during germination [67]. In pochote, the early up-regulation of genes related to spliceosome was detected mainly in the first stage of imbibition [61].

Gemination is highly regulated by the phytohormones ABA and GA3, which have antagonistic roles during this process. During germination, water uptake promotes the expression of genes related to ABA catabolism and to GA3 synthesis. The transcripts that are upregulated during seed imbibition include ABA hydrolases, that inactivate ABA, and GA oxidases, involved in GA3 synthesis [4]. 

The proper timing of germination is critical for the successful propagation and survival of seed plant species and is determined by external and internal factors. A molecular network that integrates different environmental stimuli, such as light or cold, with hormone signaling, mainly GA3, that result in the expression of cell wall proteins involved in germination was described in Arabidopsis [68]. In recent years, molecular studies of seed germination have been carried out with plants that require factors other than those described for model plants. Light is a fundamental factor that regulates germination as it provides information related to the photoperiod, the season of the year, the position of the seed in the soil and the presence of competitors. In general, light is perceived by phytochromes which regulate hormone levels. In Arabidopsis, red light induces the expression of GA biosynthesis genes and ABA degradation genes to induce germination, whereas the expression of the genes responsible for the degradation of GA and the synthesis of ABA decrease. The *Aethionema arabicum* Blum. (stonnecress) is an annual plant that presents accessions with different responses to light. In particular, the germination of the CYP accession is inhibited by light. Transcriptomic studies carried out in stonnecress showed that inhibition by light involves the same regulatory elements described for a positive response to light in Arabidopsis. In the CYP accession, light promotes the synthesis of ABA and increases the degradation of GA [69].

The seeds can have an associated microbiota (mainly fungi and bacteria). The endophytic microbiota is present in the internal tissues of the seeds and is transmitted from the mother plant to the seeds and from the seeds to the seedlings. The epiphytic microbiota, found on the surface of the seeds, are obtained by their interaction with the surrounding environment after dispersal, which generally occurs by horizontal transmission. The endophytic microbiome is often distinct from the microbiome associated with the soil in which the plants have grown [70,71]. Microbiome diversity can vary from species to species, genotype to genotype, geographic location, and the presence of other microorganisms. However, endophytic bacteria can be highly conserved in some plant species [70,71,72,73].

Seed microbiome can directly impact their quality, influencing biomass accumulation, the production of metabolites, desiccation tolerance, and resistance to pathogens. The microbiome also can promote germination by releasing seeds from dormancy through cytokinin production [71].

The domestication of plants is an important determinant of the microbial diversity of seeds. The globalization of the seed trade could lead to the homogenization of the seed microbiome on a global scale. To what extent and how domestication affects the microbiome of seeds is something that needs to be studied [74,75].

In the Orchidaceae family, seed germination is promoted by mycorrhizae colonization. Orchid seeds are very small and have very few reserves, so colonization with a compatible fungus is essential for the seed-seedling transition. In the orchid *Anoectochilus roxburghii* (Wall.) Lindl. (jinxianlian), a transcriptomic analysis was carried out to determine the effect of symbiosis on the development of the first true leaf, defined by the authors as the culmination of germination. The results obtained suggested that the interaction with the fungus induced the expression of genes related to the GA signaling pathway, promoting germination [14]. For the orchid *Dendrobirum officinalis* Kimura and Migo (gold plant), the level of transcripts related to ABA and jasmonic synthesis was lower during the first true leaf stage from colonized seeds compared with non-colonized seeds [76]. In another study performed on gold plant, the colonized seeds required less time to reach the testa rupture stage with respect to the control seeds. This advancement in the timing of the transition to the other physiological stages was also observed between the colonized and non-colonized seeds. At the testa rupture stage, when comparing the molecular profile, it was concluded that the germination process was not affected by the symbiosis with the fungus, although it did have a positive influence on the subsequent developmental stages, the protocorm and first true leaf development [64]. In general, the three studies describe that the symbiotic interaction between mycorrhizal fungi and orchid seeds induce higher expression of some key proteins involved in lipid and carbohydrate metabolism, and thus improves the efficiency of utilization of stored substances present in the embryo [14,64,76].

**Figure 1:**
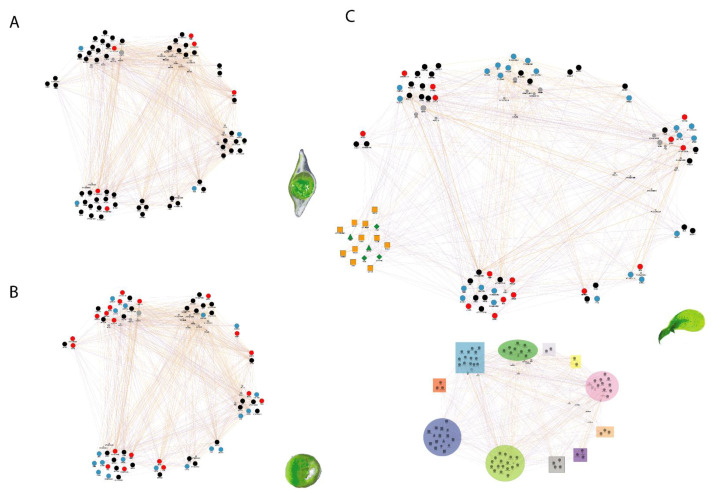
Network comparison between symbiotic germination (up-regulated) and asymbiotic germination (down-regulated) in three different developmental stages in *Dendrobium officinale*. The network was constructed using the differential expressed genes reported by Chen et al. [64] using the geneMANIA app (v3.5.2; [77]) in Cytoscape (v3.8.2; [78]) predicting the function on Arabidopsis gene sets using the information reported in Ronemus et al. [79] and Lee et al. [80]. (**A**) Stage 2: testa rupture (germination). (**B**) Stage 3: protomeristem appearance (protocorm). (**C**) Stage 4: emergence of the first leaf (seedling), at this stage we incorporate the phytohormonal regulation described by Wang et al. [76]. In the bottom left corner we present the color guide of the functional clustering: red, inorganic ion transport; blue, posttranslational modification; dark green, translation and ribosomal structure and biogenesis; lilac, lipid transport and metabolism; yellow, coenzyme transport and metabolism; pink, carbohydrate transport and metabolism; orange, nucleotide transport and metabolism; purple, amino acid transport and metabolism; gray, energy production and conversion; pale green, unknown/general function prediction; and dark blue, phytohormonal regulation (upregulated, orange, and downregulated, green; squares, SL regulators; triangles, JA regulators; and diamonds, ABA regulators).

Figure 1 shows the resulting network from the co-expression and predicted relations between the differentially expressed genes reported by Chen et al. [64] and Wang et al. [76]. In accordance with Chen et al. [64], the expression is similar under asymbiotic and symbiotic seeds during testa rupture (Figure 1A) and major changes begin during the protocorm formation stage (Figure 1B). In stage 3, the fungal interaction induced a higher and earlier expression of several disulfide-isomerase and Cucumisin proteins involved in the posttranscriptional process (blue cluster), and a higher expression of 6-phosphogluconolactonase 4 or Beta-D-xylosidase 4, enzymes related to carbohydrate metabolism (pink cluster). Additionally, the downregulation of genes, such as 2,3-bisphosphoglycerate-independent phosphoglycerate mutase and Enolase 2, was described as improving the efficiency of the utilization of stored substances.

At the seedling stage (first leaf, Figure 1C), Chen et al. [64] reported a downregulation of the activities in translation (green), and amino acid transport (purple), and metabolism pathways (present in different clusters). An upregulation of four genes related to photosynthesis was detected at the first leaf stage (pale green), when the transition from heterotrophy to photoautotrophy occurs. In the work presented by Wang et al. [76], they described phytohormonal regulation at the first leaf stage, thus their data were included in the network for this stage (Figure 1C). An important downregulation for ABA and JA pathways occurred in the first leaf from the symbiotic germinated seeds. In contrast, the SL pathway was upregulated, as it has been observed for other seed interactions. Figure 1C integrated the data from two different studies at the same physiological stage, the first leaf stage. This allows for other exploratory analyses based on predicted and confirmed genetic co-expressions in order to generate new hypothesis of the genetic regulation mediated by the fungal interaction.

About 1% of plant species are parasitic and are present in 19 families. These plants obtain resources, such as water, carbohydrates and minerals, from a host plant. Some parasitic species can present chlorophyll and carry out photosynthesis to a certain degree (hemiparasites), while others do not (holoparasites; [81]). In both types of parasitism, germination is characterized by the requirement of a molecular recognition signal from the host, so that the interaction can take place [82]. This molecular signal is given, mainly, by strigolactones (SLs) released by the host to the rhizosphere. The germination of parasitic plants was prominently studied in the Orobanchaceae family [83]. The seeds of broomrape species (*Orobanche spp*. L.) contain few reserves and can survive only for a few days after germination unless they reach a host root to establish a xylem connection. Germination in parasitic species requires a period of conditioning (priming), or an imbibition period, during which sensitivity to SLs is acquired. Without this conditioning period, the seeds cannot germinate, even in the presence of SLs or GAs [83]. Using a synthetic SL (GR24), studies suggest that SL generates an increase in the production of gibberellins and cytokinins, and a decrease in the production of ABA [83,84].

Transcriptomic changes associated to conditioning and to SL stimulation using GR24 were studied in *Phelipanche aegyptiaca* (Pers.) Pomel (Egyptian broomrape) seeds. DNA reparation, protein synthesis and carbohydrate metabolism processes were induced during conditioning. The expression levels of the GA20OX and GA3 genes were also increased during this treatment. After stimulation, a promotion of protein and carbohydrate metabolism and an important regulation of GA and BR biosynthesis was observed. For ABA, the gene NCED, involved in ABA synthesis, was down-regulated and the CYP707A1 gene, which participates in ABA catabolism, was upregulated [30]. 

## 5. Domestication, Seed Management and the Need for Rewilding Seed Science

With the rise of human settlements across the world, so began the management of plants through the domestication process about 12,000–6000 years ago [85]. To date, it is estimated that about 1000–2500 plant species around the world, belonging to 120-160 taxonomic families, have undergone some level of domestication or management by humans [86]. The spread of crop species outside of their wild ancestors original distribution range is a statement of a successful plant-animal mutual relationship that has increased the fitness of domesticated plants [87]. Several traits have evolved during domestication, that are distinguishable between the cultivated plants from their wild ancestors [85]; an example of this divergence in traits can be observed between the cultivated wheats and their wild ancestors in the genus Aegilops [88]. The term “domestication syndrome” was initially used to describe traits described in crop cereals, but, in recent years, its use has expanded to other domesticated species [89]. Many traits present in domesticated plants show signs of convergence across taxa, but the subjacent mechanisms from which they arose are part of the specific taxa, cultivar or landrace evolutionary history [85]. In this section, we will also refer to “domesticated plants” and to plants that have had some sort of management, selection or cultivation by humans as “managed” plants, in order to distinguish them from true wild species. This distinction is important, managed species have a wild origin, but in practice they can be subject to selection, breeding or variability reduction that does not necessarily reflect the original diversity of the population. These practices include the selection of a single plant or the “single seed descent” for subsequent seed production [90,91].

Seed research has depended heavily on crop and model species, but it is known that there are several seed characteristics that show signs of “selective sweeps” or a domestication signature. There has been a particular interest in fast and uniform germination, better germination in increased soil disturbance or depth in the soil, increased seed size and reduced seed shattering [87]. Seed traits are majorly selected at the onset of the domestication history of a species [85,86]. In Table 1 we present some of the representative seed-related genes with these signatures, and the varied molecular mechanisms that generated the change (from punctual mutations to loss of function, to changes in regulation). The frequency of the major crops of the Poaceae family is noticeable, although this could be partially explained by the historical importance of said family. Still, some of these genes have also been reported in members of other families, especially the Brassicaceae and the Fabaceae. Thus, it is evident that there are still unexplored areas of seed science that could benefit from taking into consideration that many of the seed traits observed in the majority of the managed species might have been subjected to systematical (if unconscious) selection at some point. Additionally, as reported in a review by Kilian and Graner [90] about the status of ex situ seed banks across the globe, there is a clear need for turning efforts towards the collection of wild species, since about 50% of the total seed accessions stored in about 1700 seed banks correspond to only 10 major crop species with multiple, redundant accessions. Aside from the cultivated species present in germplasms, there has been an increasing interest in the ex situ conservation of crop wild relatives (CWR), as they are useful in breeding programs to enhance crop production and resistance to environmental factors [88,92]. Still, these efforts only amount to about 4% of European accessions present in ex situ seed banks. This estimate reflects that, despite the potential benefits of CWRs in crop management, and even their ecological importance, wild species are profoundly neglected from conservation efforts, both ex situ and in situ [88,92]. Ex situ conservation efforts pose a challenge with no straight-forward solutions, and, for the case of CWRs, several strategies have been proposed to prioritize and focus conservation efforts [88,92], but these strategies should be implemented and adjusted for the other non-crop-relative wild species, as they could fall even far behind in the priority listings. Pedrini and Dixon [91] propose a set of standard practices that can be implemented for the specific handling of wild seeds, which include prioritizing population diversity and variability, key elements to tackle environmental crises, such as the restoration of disturbed habitats and species adaptation to climate change.

Another emerging line of evidence that should be considered in the decision of studying the germination process in wild species, and their encompassing variability, comes from whole genome and transcriptome analyses through next generation sequencing technologies. In rapeseed, the expansion and diversification of spring, winter and semi-winter cultivars is tightly associated with the selection of the genetic network that controls the responses to stress and developmental processes associated to flowering time [93]. As shown in previous sections, seed development and maturation (as well as proper timing and synchronization of developmental switches, such as flowering time) are fundamental for the success of germination and seedling establishment. In recent years there has been an increasing interest in comparative transcriptomic analyses between a domesticated crop and its wild relatives to improve breeding programs. In a study performed in *Zea mays* L. (maize) and *Zea mexicana ssp. Parviglumis* (H. H. Iltis and Doebley) Greb. (teosinte), it was demonstrated that domestication has caused a considerable reshaping of the maize transcriptome in comparison to teosinte. This reshaping also caused an important reduction in the complexity of the genetic network of maize [94]. A similar result was obtained in a comparative study in tomato and its wild relatives [95], in which both transcriptome reshaping and reduction of complexity impacted in important process related to the response to environmental stimuli and stress tolerance. Although none of these studies have focused on seed germination, these studies are a statement of the impact that human management has had on plant phenotypes and environmental responses. Thus, it is important to consider the importance of these comparative studies that include wild relatives, to plan and perform seed-oriented analyses to test the extent and real impact of global genomic regulation changes on germination traits and performance.

**Table 1:** Representative genes associated to seed characteristics with evidence of selection during domestication and plant management. Several genes are conserved among taxa, displaying similar functions, which were also characterized in the model plant Arabidopsis through mutant screening. Some domesticated phenotypes are products of complex and lineage/cultivar-specific domestication histories; thus, the same phenotype can arise from multiple mutations. The domestication phase refers to the moment in which such characteristic would have most-likely arisen based on the discussion presented by the authors and on the four-phase model proposed by Meyer and Purugganan [85]: phase 1 onset of domestication; phase 2 diversification; phase 3 dispersion from original distribution; and phase 4 variety improvement.

Species	Gene Name	Characteristic	Type/Function	Type of Mutation	Domestication Phase	Reference
*Brassica rapa*	TT8	Lighter-colored testa	Transcription factor	Insertion, LOF	Diversification and dispersion	[85]
*Glycine max*	GmG	Reduced dormancy, lighter-colored testa	CAAX amino-terminal protease protein	Splicing defect, premature stop	Onset of domestication	[76]
*Oryza sativa*	qSD7-1/qPC7	Dormancy	Transcription factor	Deletion	Onset of domestication	[96,97]
*Oryza sativa*	Sdr4	Reduced dormancy	Not characterized	Insertion	Onset of domestication	
*Oryza sativa*	OsG	Reduced dormancy	CAAX amino-terminal protease protein	Non-synonymous SNP	Onset of domestication	[76]
*Oryza sativa*	GIF1	Reserve accumulation	Cell wall invertase	Cis-regulatory, restricted expression	Onset of domestication	[98]
*Oryza sativa*	Bh4	Seed color	Amino acid transporter	Deletion/LOF, premature stop	Onset of domestication	[99]
*Oryza sativa*	GS3	Seed size and weight increase	Transmembranal protein	Premature stop	Diversification and dispersion	[17]
*Oryza sativa*	OsGRF4	Seed size increase	Transcription factor	Mutation in regulator target site	Variety improvement	[100]
*Oryza sativa*	GS5	Seed size increase	Serine-carboxypeptidase	Indels, cis-regulatory	Diversification and improvement	[101]
*Oryza sativa*	GW2	Seed size increase	E3-ubiquitin ligase	Deletion, premature stop	Unknown	[15]
*Oryza sativa*	GW6a	Seed size/weight increase, plant biomass	Histone H4-acetyltransferase, transcription regulator	Cis-regulatory	Variety improvement	[102]
*Oryza sativa*	Ae1	Amylose properties	Alpha-amylase	SNPs	Onset of domestication	[103]
*Panicum miliaceum*	PmGBSSI	Starch properties	Granule-bound starch synthase	Deletion, LOF, frameshift, missense	Diversification and dispersion	[85]
*Setaria italica*	WAXY	Starch properties	Granule-bound starch synthase	Insertion LOF	Diversification and dispersion	[104]
*Solanum lycopersicum*	SolyG	Reduced dormancy	CAAX amino-terminal protease protein	Non-synonymous SNP	Onset of domestication	[76]
*Triticum aestivum*	WAP2 (Q)	Reduced seed shattering, plant height and other pleiotropic effects	Transcription factor	Missense, cis-regulatory, premature stop	Onset of domestication	[105]
*Zea mays*	Bt2 (SSU)	Amylopectin properties	ADP-glucose pyrophosphorylase	Signal peptide, expression site	Onset of domestication and diversification	[106] and references therein
*Zea mays*	Vgt1	Flowering time QTL	AP2-like transcription factor	Cis-regulatory	Diversification and dispersion	[107]
*Zea mays*	HEX9	Glycolysis pathway, trans eQTL effects	Hexokinase 9	Cis-regulatory	Not determined	[76]
*Zea mays*	Adh2	Resistance to hypoxia	Alcohol dehydrogenase	Small tandem repeats, indels	Onset of domestication	[108]
*Zea mays*	Su1	Starch biosynthesis, sweetness	Isoamylase	Insertion LOF, missense, premature stop	Onset of domestication	[108]
*Zea mays*	DULL1	Starch synthesis	soluble starch synthase	Insertion, reduced expression	Diversification	[94]

In recent years, the number of “omics” studies performed in non-model species have increased considerably. In this review we selected some representative studies to reflect this new approach to the molecular bases of seed dormancy and germination, as well as the seed-seedling transition. In Table 2 we summarize the main characteristics of the species presented in this review, focusing on life history and habitat, storage behavior, dormancy, and management status. Of the 31 species reported, 10 correspond to wild species which reflect a wide diversity of families, habitats, life histories, storage behavior and dormancy types. This contrasts to the pattern observed for the model species, which usually belong to a few families, present orthodox seeds with physiological dormancy, come from temperate climates and have an herbaceous, annual life history. Nine species also belong to the monocot clade, but five of them are in fact from the Poaceae family. It will be important to include a wider variety of families and genera within this clade to have a better understanding of the germination process in the monocot clade.

## 6. Concluding Remarks

Climate change and ecosystem deterioration are the major challenges to tackle in the coming years. As mentioned by various authors presented in this review, our capacity to ameliorate and delay the progression of these crises will depend on the understanding of the mechanisms that allow organismal adaptation and responses to the environment. Germination in non-model species represents a challenge for their study due to the wide genetic and physiological variability they present, which must also be conserved in order to face climate change. In seed maturation studies, physiological age is used to study and compare the processes between different species and maturation conditions. Considering the great variability in the germination performance of non-model species, the establishment of morphological parameters and physiological stages is essential. These approaches are exemplified for the cases of evergreen oak, poplar, pochote and gold plant. Likewise, these parameters allow the integration of the information generated by different authors for the same species, since the definition of germination may vary. To date, most of the characterization of developmental switches and phase transitions related to dormancy and germination have been studied in different model species, such as Arabidopsis. Still, the physiological/developmental-stage approach used in many of these studies can be implemented in wild species to explore the relevance of the signaling cascades reported for Arabidopsis and other systems. This characterization will be fundamental to understanding dormancy mechanisms, aside from the non-deep physiological dormancy that is displayed by the majority of the model plants studied to date.

The domestication process has reduced the diversity and complexity of genetic interactions in the species studied. Therefore, global studies that include wild species are needed to be able to study germination in relation to its interaction with the environment. Transcriptomics and proteomics comparisons across species will help provide a better understanding of seed-related processes, such as maturation, the transitions needed for commitment to germination, as well as seedling establishment. This knowledge will also provide a better approach for breeding programs and/or in biotechnological studies to improve crop yields, as well as adaptation to climate change.

## Figures and Tables

**Table 2:** Origin and management of the studied species reported in this review. Abbreviations: D, dicotyledons; G, gymnosperms; M, monocotyledons; Cu, cultivated; Do, domesticated; Ma, managed; W, wild; PHS, preharvest sprouting.

Name	Family	Clade	Manage-ment	Original Climate	Life History	Storage Behavior	Dormancy
*Aconitum heterophyllum*	Ranunculaceae	D	W	Sub-alpine	Herbaceous, perennial	Possibly orthodox	Morpho-physiological
*Aethionema arabicum*	Brassicaceae	D	Ma	Semi-arid	Herbaceous, annual	Orthodox	Morphotype-dependent, physiological
*Arabidopsis thaliana*	Brassicaceae	D	Ma	Temperate	Herbaceous, annual	Orthodox	Physiological
*Arachis hypogaea*	Fabaceae	D	Do	Alpine	Herbaceous, annual	Orthodox	Physiological
*Avicennia marina*	Acanthaceae	D	W	Subtropical	Shrub, tree, perennial	Recalcitrant	Viviparous
*Brassica rapa*	Brassicaceae	D	Do	Semi-arid	Herbaceous, annual	Orthodox	Physiological
*Castanospermum australe*	Fabaceae	D	Ma	Tropical, subtropical	Tree, perennial	Recalcitrant	Not determined
*Ceiba aesculifolia*	Malvaceae	D	W	Tropical, subtropical	Tree, perennial	Orthodox	Non dormant
*Chenopodium quinoa*	Amaranthaceae	D	Do	Temperate	Herbaceous, annual	Orthodox	PHS
*Cynara cardunculus* var. *sylvestris*	Asteraceae	D	W	Mediterranean	Herbaceous, perennial	Possibly orthodox	Physiological
*Fraxinus hupehensis*	Oleaceae	D	W	Temperate, no dry season	Tree, perennial	Not determined	Not determined
*Glycine max*	Fabaceae	D	Do	Subtropical	Herbaceous, annual	Orthodox	Non-dormant
*Lactuca sativa*	Asteraceae	D	Do	Mediterranean, temperate	Herbaceous, annual	Orthodox	Physiological dormancy
*Medicago truncatula*	Fabaceae	D	Do	Mediterranean	Herbaceous, annual	Orthodox	Physical
*Phelipanche aegyptiaca*	Orobanchaceae	D	W	Temperate, desertic	Herbaceous	Not determined	Not determined
*Populus nigra x Populus simonii*	Salicaceae	D	Do	Temperate	Tree, perennial	Possibly Orthodox	Possibly non dormant
*Quercus ilex*	Fagaceae	D	Cu	Mediterranean	Tree, perennial	Recalcitrant	Non dormant
*Quercus robur*	Fagaceae	D	Cu	Temperate	Tree, perennial	Recalcitrant	Non dormant
*Nicotiana tabacum*	Solanaceae	D	Cu	Tropical	Herbaceous, annual	Orthodox	Physiological
*Solanum lycopersicum*	Solanaceae	D	Do	Tropical	Herbaceous, annual	Orthodox	Physiological
*Anoectochilus roxburghii*	Orchidaceae	M	W	Temperate, no dry season	Herbaceous, perennial	Orthodox	Not determined
*Calanthe tsoongiana*	Orchidaceae	M	W	Temperate, subtropical	Herbaceous, perennial	Not determined	Not determined
*Dendrobium officinale*	Orchidaceae	M	W	Tropical, subtropical	Herbaceous, perennial	Orthodox	Not determined
*Leymus chinensis*	Poaceae	M	Ma?	Temperate	Herbaceous, perennial	Orthodox	Possibly physiological
*Oryza sativa*	Poaceae	M	Do	Temperate and tropical	Herbaceous, annual	Orthodox	Cultivar-dependent. Physiological to PHS susceptible
*Paris polyphylla*	Melanthiaceae	M	Cu?	Temperate	Herbaceous, perennial	Not determined	Morpho-physiological
*Triticum aestivum*	Poaceae	M	Do	Semi-arid	Herbaceous, annual	Orthodox	Non-dormant, PHS susceptible
*Zea mays*	Poaceae	M	Do	Tropical	Herbaceous, annual	Orthodox	Non-dormant, PHS susceptible
*Zea mexicana ssp. parviglumis*	Poaceae	M	W	Tropical	Herbaceous, annual	Orthodox	Physiological
*Cunninghamia lanceolata*	Cupressaceae	G	Cu?	Temperate	Tree, perennial	Probably orthodox	Physiological
*Ginkgo biloba*	Ginkgoaceae	G	Cu	Mediterranean	Tree, perennial	Recalcitrant	Morpho-physiological
